# Comparison of Mortality and Postoperative Complications Between Open and Laparoscopic Repair of Perforated Peptic Ulcer: An Umbrella Review

**DOI:** 10.1155/2024/5521798

**Published:** 2024-11-09

**Authors:** Foolad Eghbali, Mahdi Banijamali, Fatemeh Jahanshahi, Adnan Tizmaghz, Hamid Rezvani, Parmida Ghadimi, Ahmad Madankan, Homan Alipour, Hamed Vaseghi, Meisam Haghmoradi, Mansour Bahardoust, Hesam Mosavari

**Affiliations:** ^1^Department of Surgery, Minimally Invasive Surgery Research Center, Division of Minimally Invasive and Bariatric Surgery, Rasool-e Akram Hospital, Iran University of Medical Sciences, Tehran, Iran; ^2^Student Research Committee, Faculty of Medicine, Iran University of Medical Science, Tehran, Iran; ^3^Department of General Surgery, School of Medicine, Firoozabadi Hospital, Iran University of Medical Sciences, Tehran, Iran; ^4^Rasoul Akram Hospital, Iran University of Medical Sciences, Tehran, Iran; ^5^Faculty of Medicine, Tabriz University of Medical Sciences, Tabriz, Iran; ^6^Faculty of Medicine, Urmia University of Medical Sciences, Urmia, Iran; ^7^Department of Epidemiology, School of Public Health, Shahid Beheshti University of Medical Sciences, Tehran, Iran; ^8^Department of Surgery, Surgery Research Center, School of Medicine, Rasool-E Akram Hospital, Iran University of Medical Sciences, Tehran, Iran

**Keywords:** perforated peptic ulcer, perforated peptic ulcer repair, umbrella review

## Abstract

**Background:** Perforated peptic ulcer (PPU) is one of the common complications of peptic ulcers. Open repair (OR) is the traditional surgical treatment for this condition, but with advances in laparoscopic and minimally invasive surgery, laparoscopic repair (LR) has gained popularity. Many studies have compared the effectiveness of OR vs. LR for PPU. However, the superiority of one method over the other remains a topic of debate. We conducted this review to investigate the advantages and disadvantages of LR over OR.

**Methods:** PubMed, Scopus, Google Scholar, and Web of Science were searched from 2000 to 2022 for systematic reviews and meta-analyses comparing OR and LR for PPU. Previous studies included seven postoperative outcomes, including mortality, reoperation, postoperative ileus, intra-abdominal abscess, leakage, pneumonia, and wound infection. Two researchers independently extracted data and assessed the quality of the eligible studies using the AMSTAR 2 tool.

**Results:** Five systematic reviews and meta-analyses were included, involving 36 studies. The pooled estimate showed that the risks for mortality, postoperative ileus, and wound infection were significantly lower with LR. In comparison, the risks for reoperation and leakage were less with OR. Based on the pooled estimate, no significant relationship was noted between the surgical method and intraabdominal abscess or postoperative pneumonia.

**Conclusion:** Evidence suggests that in stable patients with PPU, LR is better than OR in terms of mortality. However, more high-quality evidence is needed to determine which is more appropriate for different circumstances (e.g., unstable or high-risk patients).

## 1. Introduction

Our knowledge about peptic ulcer disease (PUD) pathogenesis has significantly evolved in the past two decades. The successful eradication of *Helicobacter pylori* with medical treatment and prophylactic use of antacid medications (e.g., proton pump inhibitors [PPI] and H-2 receptor blockers) has drastically decreased the disease incidence [1–3]. Still, the incidence of perforated peptic ulcer (PPU), one of the major complications of PUD and a surgical emergency with an associated mortality of 30%, has not significantly decreased [4–6].

PPUs can be managed nonoperatively or with abdominal surgery (either open or laparoscopic). Current recommendations on nonoperative management are to consider them in a sealed perforation, confirmed with a water-soluble contrast study. Generally, operative treatment is recommended for PPUs and should be done as soon as possible [5, 7, 8].

Open abdominal surgery has been a traditional treatment for PPU. However, laparoscopic repair (LR) for PPU, first reported in 1989 by Mouret [9], has gained popularity in recent decades. Laparoscopic surgery has some advantages over open surgery, including better-magnified visualization, minimal incision, less postoperative pain, and faster resumption of activity and ambulation. It should be noted that the open repair (OR) procedure still has advantages, including the opportunity of direct tactile sense, shorter operative time, and more effective peritoneal cavity flushing. Although OR is more accessible for young surgeons to master, many surgeons have opted to use the laparoscopic procedure [1, 8].

Many studies have compared the effectiveness of OR vs. LR for PPU. However, the superiority of one method over the other remains a topic of debate. Several systematic reviews and meta-analysis studies have been published to resolve this dispute. The first meta-analysis, published in 2004, revealed that LR granted superior short-term benefits only regarding postoperative pain and wound morbidity [4]. In 2005, a meta-analysis by Lunevicius and Morkevicius showed that LR had the benefits of less analgesic use, shorter hospital stay, lower risk of wound infection, and lower mortality rate but longer operating time and higher risk for suture-site leakage [10]. A meta-analysis by Antoniou et al., published in 2013, suggested that the two approaches had similar morbidity, mortality, and reoperation rates [11]. A meta-analysis by Zhou et al., published in 2015, indicated that LR is associated with lower rates of postoperative complications and in-hospital mortality [1]. Tan et al. conducted a meta-analysis published in 2016, which reported no significant difference between LR and OR in terms of overall postoperative complication rate, mortality, and reoperation rate [2]. In 2018, a meta-analysis by Zhang et al. concluded that the overall curative effect of LR is better than that of OR [12].

This umbrella review aims to investigate the advantages and disadvantages of OR compared with LR, searching between the findings of high-quality systematic reviews and meta-analyses. Umbrella reviews, synthesis of current systematic reviews, and meta-analyses are excellent tools for extensively exploring the evidence base and examining earlier studies' conflicting findings.

## 2. Methods and Materials

### 2.1. Study Design

We conducted an umbrella review involving all the meta-analyses and systematic reviews that examined the outcomes of OR vs. LR of PPU from 2000 to 2022. We followed the Preferred Reporting Items for Systematic Review and Meta-Analysis (PRISMA) guidelines [13]. In addition, the PRISMA diagram was used to show this project's input and output studies.

### 2.2. Sources

**Figure 1:**
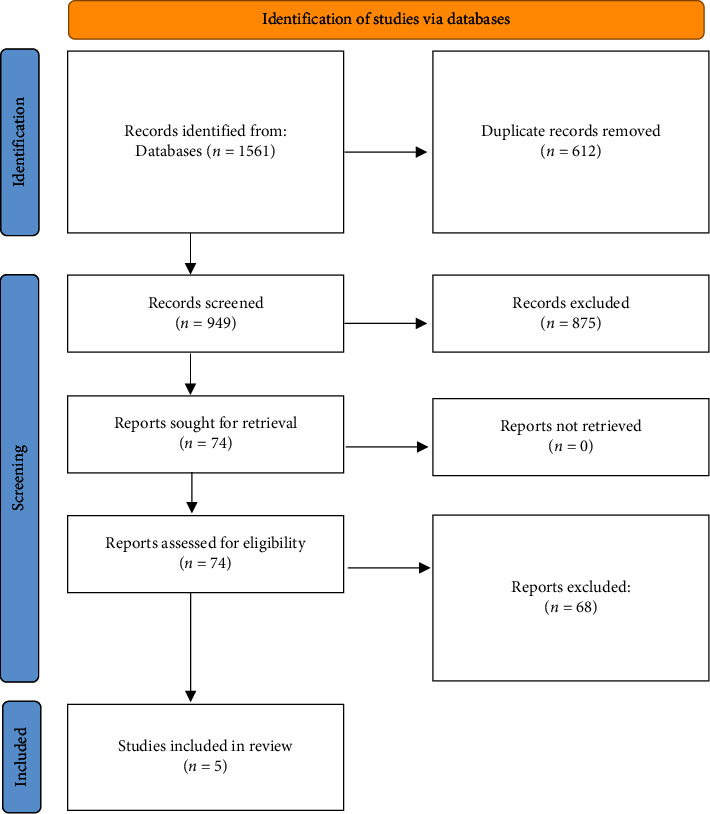
Flow diagram of study.

A comprehensive search (Figure 1) was conducted by two independent researchers. To find articles, after specifying the search strategy, PubMed, Scopus, Google Scholar, and Web of Science databases were explored to select relevant articles. The last search was done on June 20, 2022. The search concerned meta-analyses, pooled analyses, systematic reviews, and qualitative reviews on outcomes of surgical treatments, either OR or LR, for PPU. Descriptive reports, comments, letter to editor, and studies in languages other than English were excluded.

The search was conducted using the following keywords: (Omental Patch Closure OR simple Closure OR repair OR laparoscopic OR Minimally invasive OR primary repair) AND (peptic ulcer perforation OR perforated peptic ulcer OR gastroduodenal ulcer OR gastric ulcer OR stomach ulcer OR duodenal ulcer OR gastrointestinal bleeding OR gastrointestinal hemorrhage) AND (meta-analysis OR systematic review OR pooled OR overview). Search strategies used to explore PubMed, Web of Science, and Scopus databases are reported separately in Supporting 1.

### 2.3. Eligibility Criteria and Data Extraction

This study included all meta-analyses and systematic reviews of observational or clinical trial studies that evaluated the postoperative outcomes of surgical treatment for PPU. Seven outcomes were examined in this study, including wound Infection, pneumonia, leakage, intraabdominal abscess, postoperative ileus, reoperation, and mortality.

Eligibility of studies was evaluated by screening titles and, if necessary, by reviewing abstracts. Later, the full texts were assessed according to the inclusion and exclusion criteria. Studies that met the inclusion criteria were included in the study. Exclusion criteria included studies published in a language other than English, narrative or descriptive reviews, laboratory or animal studies, meta-analyses of genetic associations, and lack of access to the full text of the article. We examined all meta-analyses for associations if there were multiple meta-analyses on the same topic and outcome.

After searching, duplicate items were removed, and articles were screened to find related studies. The reference section of the articles was also examined to find more relevant studies. Two researchers independently removed the duplicate studies based on the title and abstract and finally identified the studies based on the eligibility criteria. All required information, including the study design, year of study, authors' names, type of surgical intervention, duration of follow-up, number of patients studied, number of reviewed studies, effect estimate, 95% confidence interval (CI), type of outcome, the prevalence of the outcome in subgroups, *p* value, heterogeneity, and quality of studies were extracted. In case of any conflict over a variable, the dispute was resolved by discussion between two researchers. In addition, we extracted the raw datasets of each study for reanalysis.

### 2.4. Statistical Analysis

We first analyzed the results of each meta-analysis on the relationship between surgical technique and different surgical outcomes. If there were overlapping meta-analyses, we pooled all individual study datasets from eligible meta-analyses based on the type of surgical procedure, outcome, or study design. We then reanalyzed the data after removing overlapping individual studies. Due to the lack of homogeneity of studies in terms of sample size for study subgroups, for each study and each outcome, we presented new results with effect size, 95% CI, and *p* value with random effects. All data were analyzed using STATA Version 17 software. We reanalyzed individual studies and estimated summary effects and 95% CI using randomized methods for each meta-analysis. We also calculated and presented 95% prediction intervals (PIs) for the odds ratio of the occurrence of each postoperative outcome, which measures the CI of the average accuracy of the estimate; the narrower this interval, the higher the actual accuracy. A value of *p* < 0.05 was considered significant. I2 and Cochran's Q tests were used to check the heterogeneity of the studies. Subgroup analysis was used to examine the causes of heterogeneity of variables such as quality, sample size, geographic region, and outcomes. A sensitivity analysis was performed to check the certainty of the results. Egger's test was used to check publication bias. Study results for each outcome are reported in Supporting 2.

### 2.5. Quality Assessment

The methodological quality of the studies was evaluated using the AMSTAR 2 tool [14]. All the meta-analyses that met the criteria of this article were assessed independently by two researchers. According to this tool, the quality of studies was classified into four levels: critically low (studies with more than one basic defect with or without noncritical weaknesses), low (a critical defect with or without noncritical weaknesses), moderate (more than one noncritical weakness), and high (no more than one noncritical weakness). If there was a disagreement about a study, the disagreement was resolved by consensus between the authors.

### 2.6. The Criteria for Determining the Level of Evidence

We assessed the level of evidence for each reanalyzed meta-analysis or pooled meta-analysis for the strength of associations between surgical outcomes and type of surgery (e.g., OR or LR). The criteria for determining the level of evidence were based on the analysis findings, including statistical significance with a random *p* value and 95% CI. Heterogeneity among the studies was classified based on the I2 index.

The levels of evidence based on the results were as follows.

• Convincing evidence: There was a strong statistical association for random effects between postoperative outcome and surgical method with a *p* < 0.001. The 95% CI did not include zero. There was no significant heterogeneity and no small study effect. There was concordance between the results of the most extensive study and the meta-analysis.
• Suggestive evidence: If the *p* value exceeded the significance threshold for random effects estimation (*p* < 0.05), the 95% CI included zero, but the heterogeneity between studies was not large, and there were no small study effects.
• Weak (probable) evidence: If the *p* value exceeded the significance threshold for random effects estimation (*p* < 0.05), the 95% CI included zero, the heterogeneity between studies was high, and there were small study effects.
• Nonsignificant Associations: We re-examined the results if the significance threshold for the random effect estimate did not cross the significance level (*p* > 0.05) even though the heterogeneity was high. Although most studies usually examine the increase in risk, the difference observed in the studies may be due to the difference in the estimation of the effect (e.g., risk factor, protective factor), or it may be due to the difference in the degree of association. If the difference was due to the degree of association, the level of evidence was reclassified and determined.

## 3. Results

After searching PubMed, Scopus, Web of Sciences, and Google Scholar, 1561 articles were extracted. Articles (*n* = 612) that were not in English, duplicate articles, letters to the editor, and comments were removed. The title and abstract of the remaining articles (*n* = 949) were screened to find relevant articles, and 875 articles were removed. Full text of 75 meta-analyses and systematic reviews were examined. Finally, five meta-analyses [2, 10–12, 15] with 122,510 patients that examined complications and outcomes of OR vs. LR for PPU were included in this umbrella review. The relationship between surgical methods (OR or LR) and seven postoperative outcomes or complications, including mortality, wound infection, pneumonia, leakage, intra-abdominal abscess, postoperative ileus, and reoperation, was assessed (Supporting 2).

### 3.1. Mortality

Five meta-analyses examined the relationship between mortality risk after surgery and the surgical method [2, 10–12, 15]. Only one meta-analysis reported a significant relationship between the surgical method and the mortality risk, and four studies did not report a significant correlation. R. Lunevicius et al. reported that the mortality risk after LR was 0.4 times that of OR (*p*=0.001) (Table 1). Pooled and the overall estimation of the results of 5 meta-analysis studies (36 observational studies or trials) in 1989 patients showed that the mortality risk after LR was significantly lower (OR = 0.5, 95% CI = 0.34–0.72, *p*=0.001) (Table 2).

### 3.2. Reoperation

Four meta-analyses examined the relationship between the risk of reoperation after surgery and the surgical method [2, 10, 11, 15]. Evaluation of these studies showed that only Lau et al. [15] reported a significant relationship between the type of surgery and the chance of reoperation (Table 1). The pooled and overall estimation of the results of four meta-analysis studies (20 observational studies or trials) in 1524 patients showed that the chance of reoperation after LR was significantly greater than OR (OR = 2.22, 95% CI = 1.29–3.83, *p*=0.001) (Table 2).

### 3.3. Postoperative Ileus

Four meta-analysis studies examined the relationship between postoperative ileus risk and the surgical method [2, 10, 12, 15]. Only one meta-analysis reported a significant relationship. In other studies, although the risk of this complication was lower with LR, the difference was not statistically significant (Table 1). Pooled and overall estimation of the results of 4 meta-analysis studies in 1932 patients showed that the risk of postoperative ileus in patients who underwent LR was significantly lower than in OR patients (OR = 0.52, 95% CI = 0.32–0.84, *p*=0.032) (Table 2).

### 3.4. Intra-Abdominal Abscess

Four meta-analyses investigated the relationship between the risk of intra-abdominal abscess after surgery and the surgical method [2, 10, 12, 15]. The evaluation of meta-analyses and the pooled results showed that although the risk of intra-abdominal abscess is slightly lower in LR, this difference is not statistically significant (Table 2).

### 3.5. Leakage

Three meta-analysis studies investigated the relationship between the risk of leakage after surgery and the surgical method [2, 10, 15]. Only the study of R. Lunevicius et al. [10] with 685 patients reported a significant relationship (Table 1). Pooled and the overall estimation of the results of three meta-analysis studies (21 observational studies or trials) in 1486 patients showed that the risk of postoperative leakage with LR was significantly greater than that with OR (OR = 2.69, 95% CI = 1.30–5.55, *p*=0.011) (Table 2).

### 3.6. Postoperative Pneumonia

Three meta-analyses investigated the association of postoperative pneumonia risk with the surgical method [2, 12, 15]. The evaluation of the studies and the pooled results showed that although the risk of postoperative pneumonia is lower with LR, this difference is not statistically significant (Table 2).

### 3.7. Wound Infection

Based on meta-analyses and pooled results, wound infection was the most common complication after surgery in OR and LR. Four meta-analyses investigated the relationship between wound infection risk after surgery and surgical methods [2, 10, 12, 15]. Two studies reported a significant relationship between the surgical method and the risk of wound infection. Although the risk of wound infection was lower with LR in the other two studies, this difference was not statistically significant (Table 1). Pooled and the overall estimation of the results of 4 meta-analysis studies (31 observational studies or trials) in 2485 patients showed that the risk of wound infection with the laparoscopic method was significantly lower than the open method (OR = 0.29, 95% CI = 0.2–0.44, *p*=0.001) (Table 2).

## 4. Discussion

The present study was a comprehensive review of studies comparing mortality and several postoperative complications of LR with OR for PPU. Our study's pooled estimate showed that mortality, reoperation, postoperative ileus, leakage, and wound infection were significantly related to the surgical method. The risks for mortality, postoperative ileus, and wound infection were significantly lower with LR. In comparison, the risks for reoperation and leakage were less with OR. Based on the pooled estimate, no significant relationship was noted between the surgical method and intra-abdominal abscess or postoperative pneumonia.

Generally, certain patients can be treated without surgery because some perforations may heal spontaneously (confirmed by a water-soluble contrast study). The widespread use of PPIs has significantly reduced the rate of surgery in cases of PPU. PPIs effectively reduce gastric acid production, alleviate the symptoms of peptic ulcers, promote ulcer healing, and prevent complications such as perforation. This underscores the importance of promptly initiating aggressive medical treatment with PPIs for peptic ulcers [16, 17]. However, emergent surgery may be necessary in cases of sepsis, generalized peritonitis, or when nonoperative management fails.

For the surgical management of PPU, the preferred surgical approach involves suturing the perforation, sometimes with an omental patch, which is considered the standard procedure for most cases. Traditionally, this surgery is conducted through a laparotomy, but as laparoscopic techniques have become more widespread and surgeons have gained expertise, a minimally invasive approach is increasingly favored. Recent studies indicate that LR is performed in about one-third of PPU cases [18]. Laparoscopy offers several advantages, including quicker recovery, shorter hospital stays, reduced postoperative pain, earlier return to normal activities, and improved cosmetic outcomes, resulting in enhanced patient recovery [1, 8]. Technological advancements in laparoscopy include the refinement of instruments such as endo-staplers and harmonic scalpels, as well as the introduction of robotic assistance, single-incision laparoscopic surgery (SILS), natural orifice transluminal endoscopic surgery (NOTES), 3D visualization, and tele-mentoring. These advancements, combined with modern anesthesia techniques, have greatly enhanced the safety and feasibility of laparoscopic procedures [19–24]. However, there are challenges associated with laparoscopic surgery in emergency cases, particularly those involving diffuse peritonitis, abscesses, and adhesions [25]. While evidence supporting laparoscopy largely comes from studies in elective surgeries [2], its advantages over open procedures in emergency settings remain uncertain. Laparoscopic surgery in emergencies is often considered too complex and is not typically recommended. Additionally, planning for laparoscopic procedures during off-hours or night shifts can be difficult due to time constraints and limited access to equipment and surgical staff [25]. Nevertheless, the potential benefits of laparoscopy for both diagnosis and treatment of acute abdominal conditions are recognized.

The most important shortcoming of LR in this study was postoperative leakage. Leakage has been noted as the main cause of reoperation, one of the reasons why reoperation rates are higher after LR [1]. LR is done either with sutures or without them (sutureless). An increased risk of suture-site leakage has been reported for both of these techniques [1, 5]. We assume this is the reason for higher leakage rates and reoperation with LR. An analysis of risk factors found that 84% of patients with a history of symptoms lasting longer than 9 h developed leakage after LR [26]. The higher incidence of leakage after LR may be attributable in part to a faulty technique of laparoscopic closure. Perfection and standardization of the laparoscopic technique for the repair of perforated ulcers is therefore necessary [15].

Our comprehensive review revealed that patients who underwent surgery with the LR procedure had a significantly lower risk of death compared to those who underwent open surgery. Specifically, the chance of death was halved in LR patients. In some studies, major medical illness, preoperative shock, and delayed presentation (more than 24 h) were identified as significant risk factors for mortality [15]. Therefore, considering the significant reduction in mortality rates with LR and its effectiveness relative to other treatment options, we recommend prioritizing LR for these patients.

One well-known effect of carbon dioxide (CO_2_) insufflation during LR is the elevation of intra-abdominal pressure and hypercarbia, which can potentially decrease cardiac output and pH levels for unstable patients or those with significant cardiopulmonary conditions [12]. Experimental animal studies have revealed that the increased intraabdominal pressure of carbon dioxide pneumoperitoneum is associated with an increased risk of bacteremia and sepsis when the duration of peritonitis exceeds 12 h [27–29]. Patients with no Boey risk factors (prolonged perforation for more than 24 h, shock on admission, and severe medical disorders) should benefit from LR [26]. However, our study did not differentiate between patient stability and the surgical method. The “open vs. laparoscopic” debate remains unsolved, but the current recommendation is that LR is preferable for stable patients, while OR is recommended for unstable patients. However, these recommendations are based on low- and moderate-quality evidence [3]. According to the 2020 guidelines from the World Society of Emergency Surgery (WSES), the choice between LR and OR in PPU depends on patient stability and the availability of laparoscopic equipment and skills. Evaluating patients for stability involves a thorough assessment of symptoms, signs, and laboratory findings, as well as using scoring systems like Rockall and Glasgow–Blatchford to gauge disease severity and guide treatment decisions [5].

The selection process for LR versus OR should be tailored to each patient's condition, considering factors such as the severity of their symptoms, medical history, and overall fitness for surgery, ensuring that all relevant information and potential risks were thoroughly discussed to facilitate an informed choice. In general, both methods have some advantages, and choosing one of them mostly depends on the skill of the surgeon, the available equipment, and the patient's condition.

### 4.1. Limitations

This study covered several postoperative complications. However, intraoperative difficulties and predictors of poor outcomes are essential factors for choosing the proper surgical treatment, which this study did not cover. Most of the studies were of acceptable quality for all outcomes based on the AMSTAR checklist. However, our study has some limitations that should be pointed out. Limitations of the study include variations in methodology, study location, and patient populations among the included studies, potentially impacting the overall findings. The severity of PPU in operated patients was often not reported, making it difficult to assess outcomes based on disease severity. Additionally, the inclusion of hemodynamically unstable patients and the lack of randomization in many studies may introduce selection bias. Surgeon learning curves and variations in surgical techniques across different centers can also influence outcomes, particularly concerning suture-site leakage rates after LR. Lastly, adequate follow-up is essential for assessing the long-term safety and efficacy of LR versus OR for PUD.

## 5. Conclusion

Our comprehensive umbrella review showed that LR is associated with a lower risk of mortality, postoperative ileus, and wound infection. In comparison, OR is associated with a lower risk of reoperation and leakage. However, this conclusion is based on studies that compared the outcomes of LR and OR in stable patients. When choosing between laparoscopic and open surgical repair, the surgeon's skill, equipment availability, patient's condition, and predictors of poor outcome should be considered. Further high-quality multicenter RCTs are needed to confirm the benefits of LR.

## Figures and Tables

**Table 1:** Summary of each meta-analysis of postoperative outcomes.

Type/author, year	Patients (*n*)	Included study types	Effect estimate (OR)	95% CI	*I*_2_ value	*p* value	AMSTAR 2
*Mortality*							
Antoniou et al. (2013)	128	3	0.36	0.1–1.31	0	0.12	Low
Lau (2004)	576	11	0.63	0.34–1.16	0	0.13	Moderate
Tan et al. (2016)	434	4	0.66	0.25–1.75	< 0.1	> 0.05	High
Lunevicius et al. (2005)	685	15	0.4	0.22–0.73	11.5%	0.001	Moderate
Zhang, Chen, and Li (2018)	166	2	0.29	0.04–1.99	0%	0.2	Moderate

*Reoperation*							
Antoniou et al. (2013)	111	2	2.02	0.33–12.66	0	0.44	Low
Lau (2004)	469	8	2.52	1.06–6.21	4.72	0.045	Moderate
Tan et al. (2016)	333	3	1.93	0.57–6.5	0	> 0.05	Weak
Lunevicius et al. (2005)	611	7	2.17	0.86–5.48	0%	0.1	Moderate

*Ileus after operation*							
Lau (2004)	344	5	0.62	0.2–1.92	0.5	0.41	Moderate
Tan et al. (2016)	434	5	0.46	0.15–1.39	< 0.1	> 0.05	High
Lunevicius et al. (2005)	578	7	0.71	0.36–1.42	0%	0.33	Moderate
Zhang, Chen, and Li (2018)	606	8	0.3	0.11–0.8	0%	0.02	Moderate

*Intraabdominal abscess*							
Lau (2004)	400	7	1.99	0.79–5.02	0	0.51	Moderate
Tan et al. (2016)	430	5	0.58	0.22–1.55	0	> 0.05	High
Lunevicius et al. (2005)	516	7	1.04	0.53–2.03	0.9%	0.91	Moderate
Zhang, Chen, and Li (2018)	461	6	0.42	0.16–1.09	0%	0.07	Moderate

*Leakage*							
Lau (2004)	367	7	1.49	0.53–4.24	2.47	0.45	Moderate
Tan et al. (2016)	434	5	2.2	0.67–7.17	0	> 0.05	High
Lunevicius et al. (2005)	685	9	4.75	2.14–10.54	6%	0.001	Moderate

*Postoperative pneumonia*							
Lau (2004)	540	3	0.79	0.38–1.62	0	0.45	Moderate
Tan et al. (2016)	549	6	0.61	0.26–1.41	< 0.1	> 0.05	High
Zhang, Chen, and Li (2018)	405	5	0.84	0.37–1.90	0%	0.68	Moderate

*Wound infection*							
Lau (2004)	384	6	0.39	0.16–0.94	4.39	0.036	Moderate
Tan et al. (2016)	549	6	0.32	0.17–0.61	—	0.023	High
Lunevicius et al. (2005)	709	8	0.38	019–0.76	0.9%	0.006	Moderate
Zhang, Chen, and Li (2018)	816	11	0.19	0.09–0.4	—	0.0001	Moderate

**Table 2:** Summary of the meta-analysis results by pooling all the datasets.

Complication	Number of studies	Number of total participants	Effect estimate (OR)	CI 95%	*p* value	*I*_2_ value (%)	Overall *p*	Quality of the evidence
Mortality	36	1989	0.5	0.34–0.72	0.001	0	0.74	Convincing
Reoperation	20	1524	2.22	1.29–3.83	0.001	0	0.99	Convincing
Ileus after operation	25	1962	0.52	0.32–0.84	0.033	0	0.58	Convincing
Intraabdominal abscess	25	1807	0.86	0.46–1.63	0.51	51.8	0.1	No significance
Leakage	21	1486	2.69	1.30–5.58	0.011	37.9	0.2	Convincing
Pneumonia	14	1494	0.75	0.47–1.18	0.44	0	0.85	Suggestive
Wound infection	31	2458	0.29	0.20–0.44	0.001	0	0.56	Convincing

## Data Availability

The data used to support the findings of this study are included in the article.

## Supporting Information

Supporting Information Supporting 1: Literature search strategy used for PubMed, Scopus, and Web of Science databases.

Supporting Information Supporting 2: Forest plots illustrating the meta-analyses conducted on seven postoperative outcomes and the combined plot for all outcomes.

## References

[1] Zhou C., Wang W., Wang J. (2015). An Updated Meta-Analysis of Laparoscopic versus Open Repair for Perforated Peptic Ulcer. *Scientific Reports*.

[2] Tan S., Wu G., Zhuang Q. (2016). Laparoscopic versus Open Repair for Perforated Peptic Ulcer: A Meta Analysis of Randomized Controlled Trials. *International Journal of Surgery*.

[3] Ding J., Liao G., Zhang Z. (2011). Meta-analysis of Laparoscopic and Open Repair of Perforated Peptic Ulcer. *Chinese Journal of Gastrointestinal Surgery*.

[4] Chung K. T., Shelat V. G. (2017). Perforated Peptic Ulcer-An Update. *World Journal of Gastrointestinal Surgery*.

[5] Tarasconi A., Coccolini F., Biffl W. L. (2020). Perforated and Bleeding Peptic Ulcer: WSES Guidelines. *World Journal of Emergency Surgery*.

[6] Enos J. Management of Acute Perforation.

[7] Di Saverio S., Bassi M., Smerieri N. (2014). Diagnosis and Treatment of Perforated or Bleeding Peptic Ulcers: 2013 WSES Position Paper. *World Journal of Emergency Surgery*.

[8] Hudnall A., Bardes J. M., Coleman K. (2022). The Surgical Management of Complicated Peptic Ulcer Disease: An EAST Video Presentation. *Journal of Trauma and Acute Care Surgery*.

[9] Litynski G. S. (1999). Profiles in Laparoscopy: Mouret, Dubois, and Perissat: the Laparoscopic Breakthrough in Europe (1987-1988). *Journal of the Society of Laparoendoscopic Surgeons*.

[10] Lunevicius R., Morkevicius M. (2005). Systematic Review Comparing Laparoscopic and Open Repair for Perforated Peptic Ulcer. *British Journal of Surgery*.

[11] Antoniou S. A., Antoniou G. A., Koch O. O., Pointner R., Granderath F. A. (2013). Meta-analysis of Laparoscopic versus Open Repair of Perforated Peptic Ulcer. *Journal of the Society of Laparoendoscopic Surgeons: Journal of the Society of Laparoendoscopic Surgeons*.

[12] Zhang H., Chen J., Jun Li Y. (2018). Systematic Review of Curative Effect between Laparoscopic and Open Repair for Perforated Gastroduodenal Ulcer. *Biomedical Research*.

[13] Selçuk A. A. (2019). A Guide for Systematic Reviews: PRISMA. *Turkish Archives of Otolaryngology*.

[14] Shea B. J., Reeves B. C., Wells G. (2017). AMSTAR 2: A Critical Appraisal Tool for Systematic Reviews that Include Randomised or Non-randomised Studies of Healthcare Interventions, or Both. *BMJ*.

[15] Lau H. (2004). Laparoscopic Repair of Perforated Peptic Ulcer: a Meta-Analysis. *Surgical Endoscopy and Other Interventional Techniques*.

[16] Kristjansdottir T. R., Sigurdsson M. I., Jonsdottir F. (2023). [The Incidence of Postoperative and Persistent Usage of Proton Pump Inhibitors Following Surgery]. *Laeknabladid*.

[17] Ali Khan M., Howden C. W. (2018). The Role of Proton Pump Inhibitors in the Management of Upper Gastrointestinal Disorders. *Gastroenterology and Hepatology*.

[18] Pereira A., Santos Sousa H., Goncalves D. (2021). Surgery for Perforated Peptic Ulcer: Is Laparoscopy a New Paradigm?. *Minimally Invasive Surgery*.

[19] Hunter J. G., Spight D. H., Sandone C., Fairman J. E. (2018). Advanced Minimally Invasive Surgical Techniques: Single-Incision Laparoscopic Surgery, Robotics, and Natural-Orifice Transluminal Endoscopic Surgery. *Atlas of Minimally Invasive Surgical Operations*.

[20] Kawashima K., Kanno T., Tadano K. (2019). Robots in Laparoscopic Surgery: Current and Future Status. *BMC Biomedical Engineering*.

[21] Basunbul L. I., Alhazmi L. S. S., Almughamisi S. A., Aljuaid N. M., Rizk H., Moshref R. (2022). Recent Technical Developments in the Field of Laparoscopic Surgery: A Literature Review. *Cureus*.

[22] Costa G., Garbarino G. M., Lepre L. (2024). Laparoscopic Treatment of Perforated Peptic Ulcer: A Propensity Score-Matched Comparison of Interrupted Stitches Repair versus Knotless Barbed Suture. *Journal of Clinical Medicine*.

[23] Pandya A., Reisner L. A., King B. (2014). A Review of Camera Viewpoint Automation in Robotic and Laparoscopic Surgery. *Robotics*.

[24] Link R. E., Schulam P. G., Kavoussi L. R. (2001). Telesurgery: Remote Monitoring and Assistance during Laparoscopy. *Urologic Clinics of North America*.

[25] Mandrioli M., Inaba K., Piccinini A. (2016). Advances in Laparoscopy for Acute Care Surgery and Trauma. *World Journal of Gastroenterology*.

[26] Lunevicius R., Morkevicius M. (2005). Risk Factors Influencing the Early Outcome Results after Laparoscopic Repair of Perforated Duodenal Ulcer and Their Predictive Value. *Langenbeck’s Archives of Surgery*.

[27] Bloechle C., Emmermann A., Treu H. (1995). Effect of a Pneumoperitoneum on the Extent and Severity of Peritonitis Induced by Gastric Ulcer Perforation in the Rat. *Surgical Endoscopy*.

[28] Evasovich M. R., Clark T. C., Horattas M. C., Holda S., Treen L. (1996). Does Pneumoperitoneum during Laparoscopy Increase Bacterial Translocation?. *Surgical Endoscopy*.

[29] Gurtner G. C., Robertson C. S., Chung S. C., Ling T. K., Ip S. M., Li A. K. (1995). Effect of Carbon Dioxide Pneumoperitoneum on Bacteraemia and Endotoxaemia in an Animal Model of Peritonitis. *Journal of British Surgery*.

