# Supplementary material for: Comparison of Mortality and Postoperative Complications Between Open and Laparoscopic Repair of Perforated Peptic Ulcer: An Umbrella Review
Source: Minim Invasive Surg. 2024 Nov 9;2024:5521798. doi: 10.1155/2024/5521798 (PMC11568887; doi:10.1155/2024/5521798)
Supplement: Supporting Information — Supporting 1: Literature search strategy used for PubMed, Scopus, and Web of Science databases. [file 5521798.f1.docx]

## Supplementary 1- Literature search strategy

This file contains the detailed search strategies used to identify relevant studies from the PubMed, Scopus, and Web of Science databases

PubMed

(Omental Patch Closure[TIAB] OR simple Closure[TIAB] OR repair[TIAB] OR laparoscop*[all] OR Minimally invasive[TIAB] OR primary repair[all]) AND (peptic ulcer perforation[mesh] OR "perforated peptic ulcer"[all] OR gastroduodenal ulcer[tiab] OR gastric ulcer[tiab] OR stomach ulcer[all] OR duodenal ulcer[all] OR gastrointestinal bleeding[all] OR gastrointestinal hemorrhag*[tiab]) AND (meta-analysis[tiab] OR meta analysis[tiab] OR systematic review[tiab] OR metaanalysis[tiab] OR metananalysis[tiab] OR pooled[tiab] OR overview[tiab])

Scopus

( TITLE-ABS-KEY ( "Omental Patch Closure" OR "simple Closure" OR laparoscop* OR "Minimally invasive" ) OR ( TITLE-ABS-KEY ( primary W/3 repair ) OR TITLE-ABS-KEY ( laparoscopic W/3 repair ) OR TITLE-ABS-KEY ( open W/3 repair ) ) ) AND ( ALL ( "peptic ulcer perforation" OR "perforated peptic ulcer" ) OR TITLE-ABS-KEY ( gastroduodenal W/3 ulcer ) OR TITLE-ABS-KEY ( gastric W/3 ulcer ) OR TITLE-ABS-KEY ( stomach W/3 ulcer ) OR TITLE-ABS-KEY ( duodenal W/3 ulcer ) OR TITLE-ABS-KEY ( gastrointestinal W/3 bleed* ) OR TITLE-ABS-KEY ( gastrointestinal W/3 hemorrhag* ) ) AND ( TITLE-ABS-KEY ( "meta-analysis" OR "meta analysis" OR "metanalysis" OR pooled OR "systematic review" OR overview ) OR TITLE-ABS-KEY ( systematic* W/3 review* ) OR TITLE-ABS-KEY ( meta* W/3 analys* ) )

Web of science

(TS=(Omental Patch Closure) OR TS=(simple Closure) OR AB=(repair) OR AB=(laparoscop*) OR AB=(Minimally invasive) OR TS=(primary repair)) AND (TS=(peptic ulcer perforation) OR TS=(perforated peptic ulcer) OR TS= (gastroduodenal ulcer) OR TS=(gastric ulcer) OR TS=(stomach ulcer) OR TS=(duodenal ulcer) OR TS=(gastrointestinal bleed*) OR TS=(gastrointestinal hemorrhag*)) AND (TS=(meta-analysis) OR TS=(meta analysis) OR TS=(systematic review) OR TI=(metaanalysis) OR TI=(metananalysis) OR TS=(pooled) OR TI(overview))
