# Supplementary material for: Comparison of Mortality and Postoperative Complications Between Open and Laparoscopic Repair of Perforated Peptic Ulcer: An Umbrella Review
Source: Minim Invasive Surg. 2024 Nov 9;2024:5521798. doi: 10.1155/2024/5521798 (PMC11568887; doi:10.1155/2024/5521798)
Supplement: Supporting Information — Supporting 2: Forest plots illustrating the meta-analyses conducted on seven postoperative outcomes and the combined plot for all outcomes. [file 5521798.f2.docx]

## Supplementary 2 - Results of meta-analysis for each and all outcomes

This file contains forest plots illustrating the meta-analyses conducted for each outcome measure.


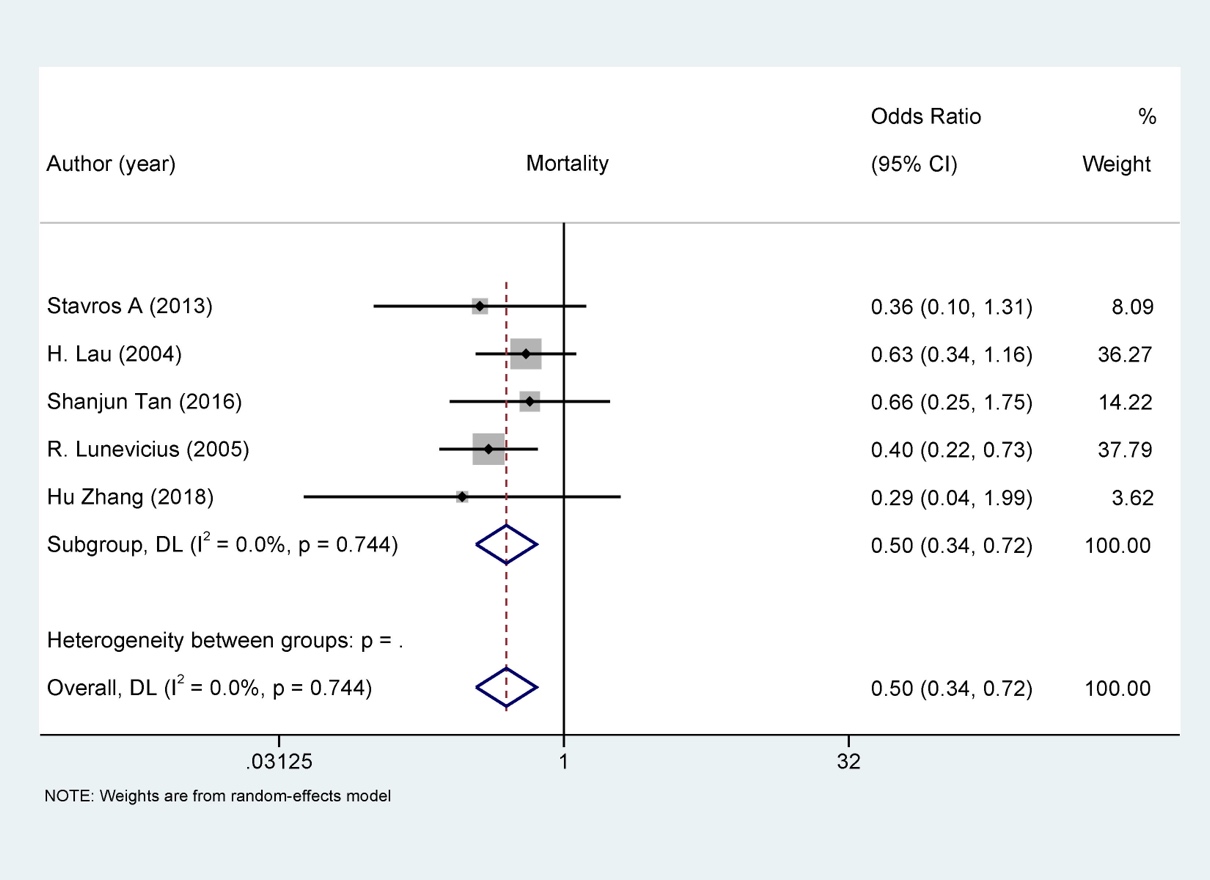


Figure S1 - Meta-analysis results for mortality


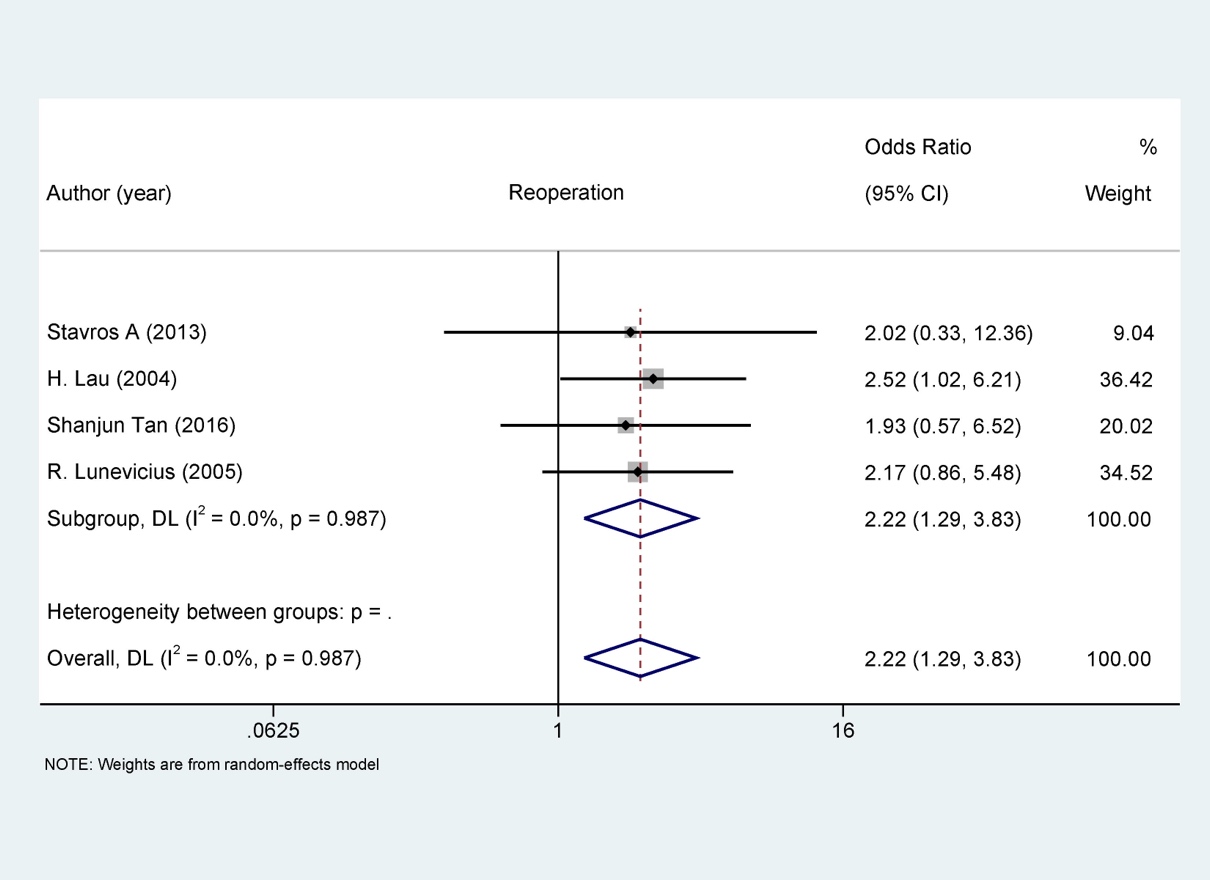


Figure S2 - Meta-analysis results for reoperation


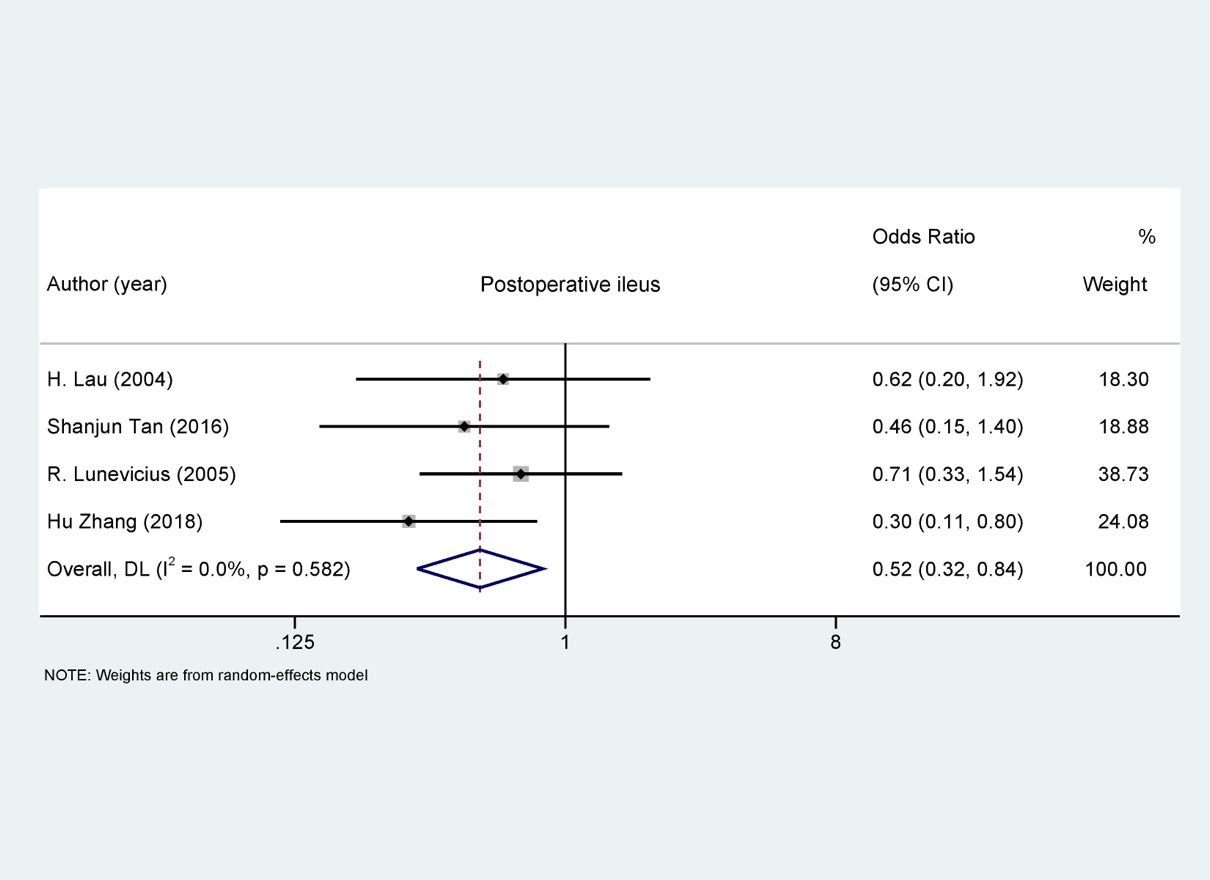


Figure S3 - Meta-analysis results for postoperative ileus


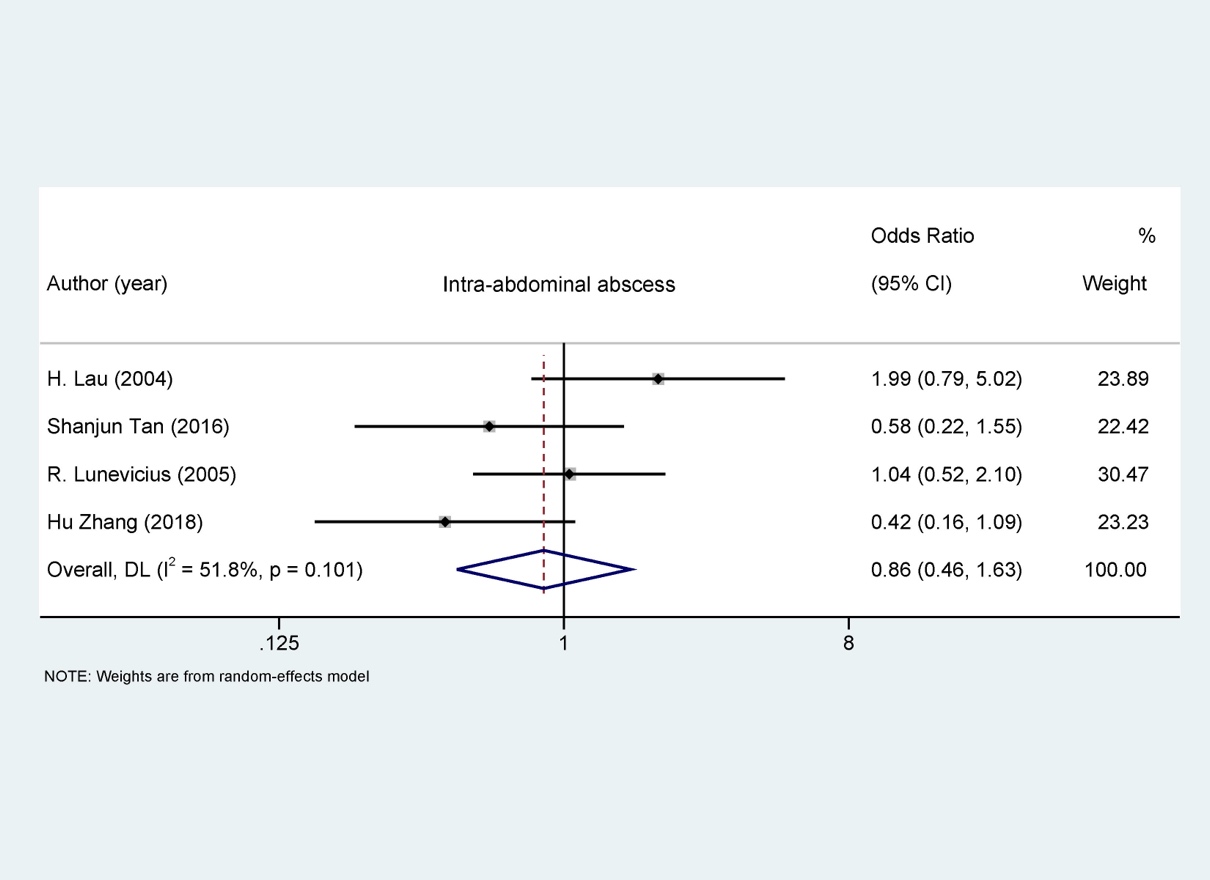


Figure S4 - Meta-analysis results for intra-abdominal abscess


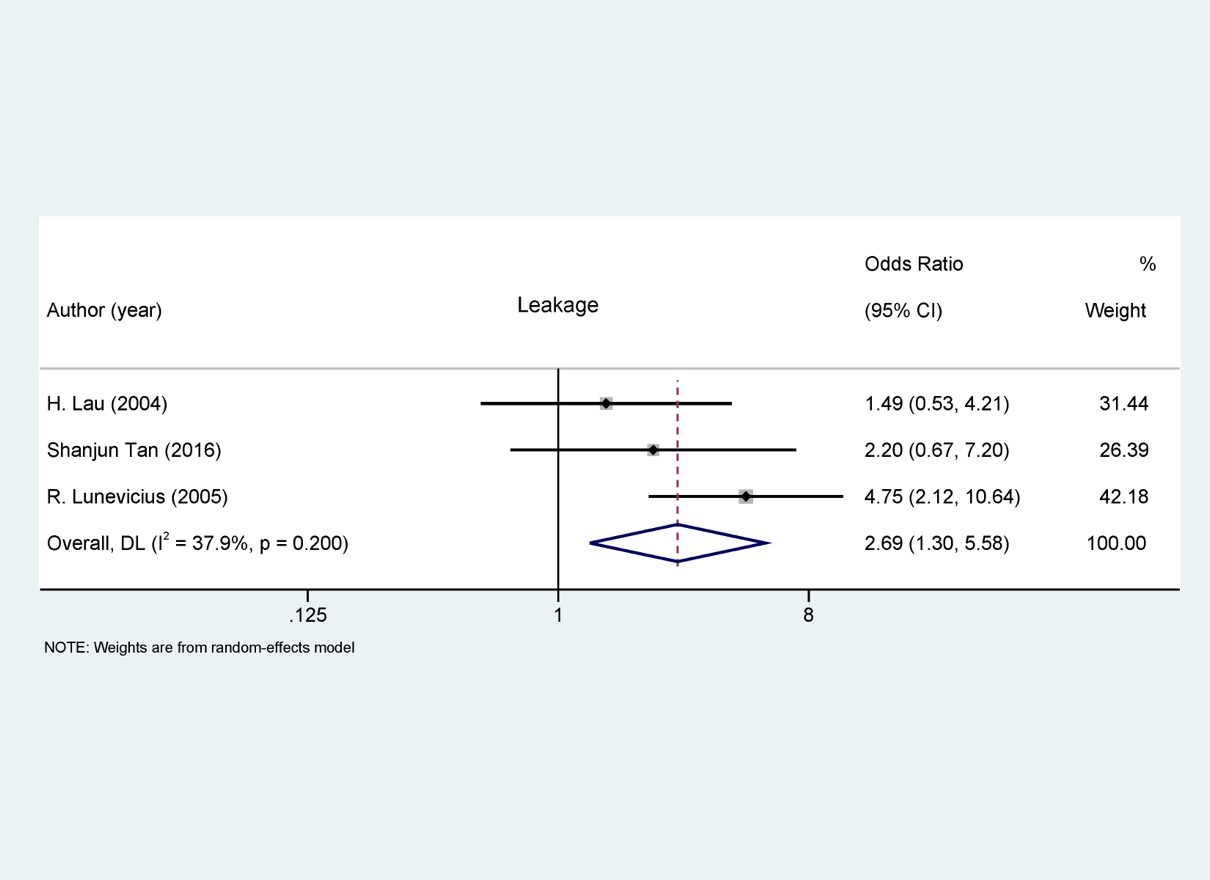


Figure S5 - Meta-analysis results for leakage


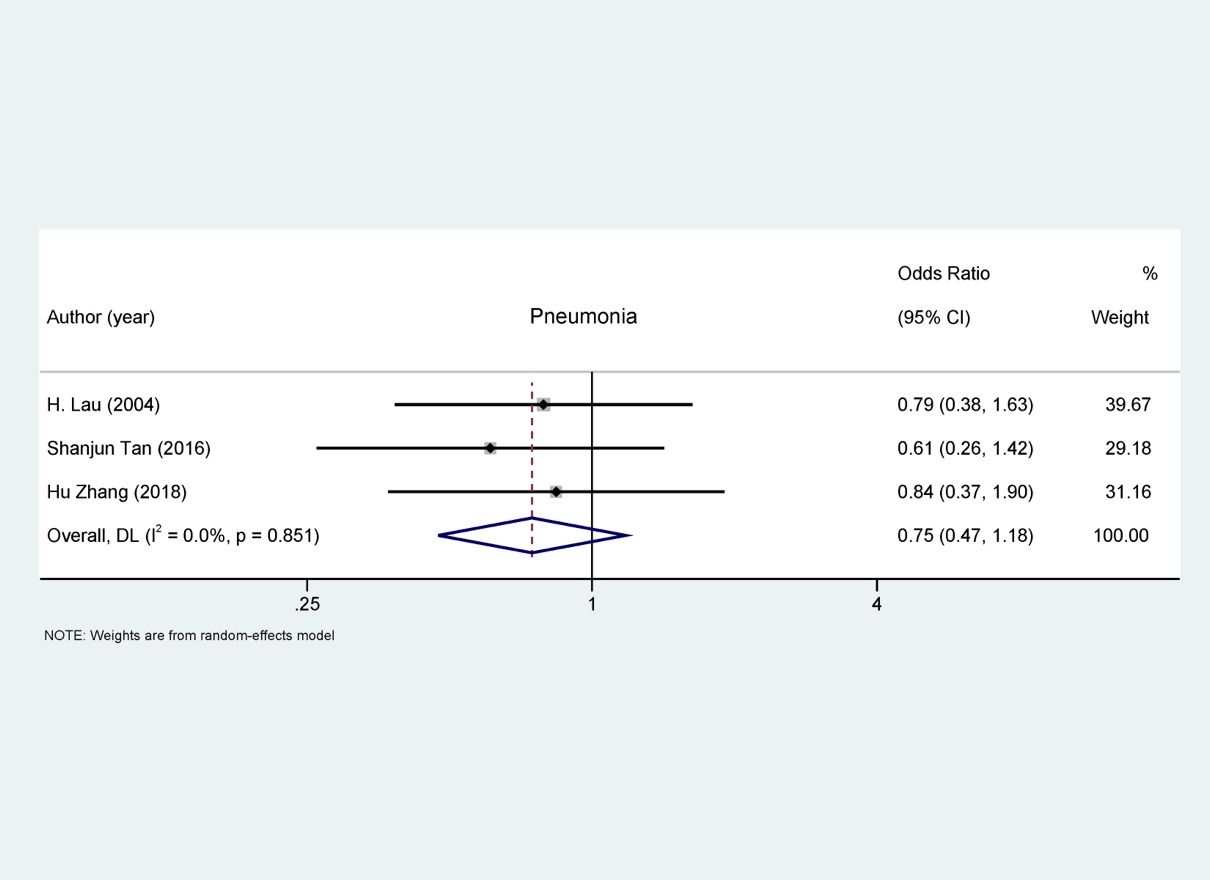


Figure S6 - Meta-analysis results for postoperative pneumonia


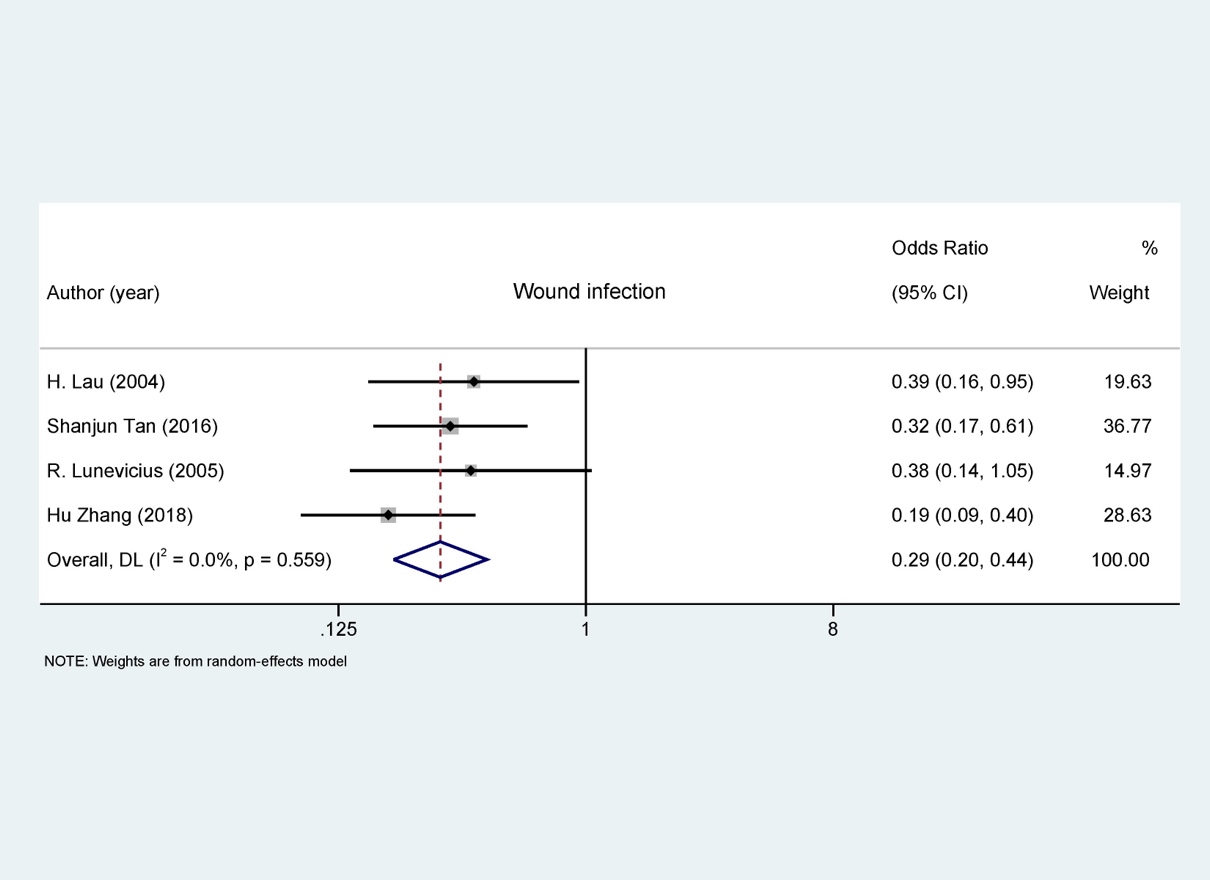


Figure S7 - Meta-analysis results for wound infection

Figure S8 - Meta-Analysis results for seven postoperative outcomes or complications, including mortality, wound infection, pneumonia, leakage, intra-abdominal abscess, postoperative ileus, and reoperation
